# Developed an Automated Design Sorting System According to Outer Edges of Object

**DOI:** 10.1155/2022/9439093

**Published:** 2022-08-02

**Authors:** Adawiya Ali Hamzah, Abbas Fadhil Abbas

**Affiliations:** ^1^Department of Energy Engineering, University of Baghdad, Baghdad, Iraq; ^2^Aeronautical Eng. Technologies Dept., Technical College of Engineering, Al-Farahidi University, Baghdad, Iraq

## Abstract

The automated sorting systems are used in the industrial sectors to increase the rate of production. This research developed the sorting system by using a vision machine to detect the matching of capturing image with the storage base image. The system will be matching and sorting in real-time with 5 cm/s conveyor belt speed. The vision system is based on three stages to arrive at the sorting decision. The first stage is to covert the capturing image to a binary image, the second stage is applying edge detection of the product, and the third stage is matching this result with the base image. The system was successful to sort any product with complexity in shape and with high efficiency. The system sorting can be detected and sorted any product/machine element at any position or orientation. The system uses real-time analysis in order to provide the required results. The results arrived at the sorting gate at the end conveyer belt of the system if open that means the product matching. Three different products were selected in order to investigate the response and the accuracy of the results. It was found that the maximum of error to detect the product is not exceeding 2% for all cases.

## 1. Introduction

The automated sorting system become an essential element in industrial sectors due to decreased time and increased rate of sorting. It was using sensors that connect to control the microcontroller or PLC for producing the right decision. The sorting method depends on the color, shape, volume, size, and type of detection products. The actuator sorting consists of an arm robot, sorting gate, and hydraulic link. The sorting process uses mainly vision computers with artificial intelligence and statistical methods.

The application of sorting systems is in all industrial sectors such as sorting fruits by size with three categories, finding the radius, and calculating the area and perimeter based on light detection and ranging (LIDAR) sensor [1]. Some researchers design the systems to sort fruits using a vision system and robot with tracking the positions [2, 3]. PLC controller is used to sort objects based on the material of the subject such as wood, steel, and plastic with three sensors at different positions with acceptable average time [4]. Machine vision adds to sorting systems for detecting and automated sorting [5–7]. Also, the image processing approach with deep learning as a system is used to sort objects [8].

The sorting of an object is based on four methods that are related to color, shape, texture, and hybrid approach [9]. The support vector machine (SVM) is utilized in some systems with a mobile manipulator in cases where the system accomplished the step tasks such as the recognition of objects at any varying scales and rotations, motion control plan for mobile manipulation, and grasping control plane for objects at any position [10].

Sorting parts and remanufacturing processes use logic cube inspection to detect the product and sort based on its weight and volume with machine vision, but the system does not work in real-time [11]. Automated defect detection by a visual inspection system for cylindrical objects is performed using a camera and multimirror handle, but such a system does not work with real-time detection [12]. Some vision systems are using robots to recognize and sort objects, and such systems sometimes use PLC control units [13, 14].

**Figure 1:**
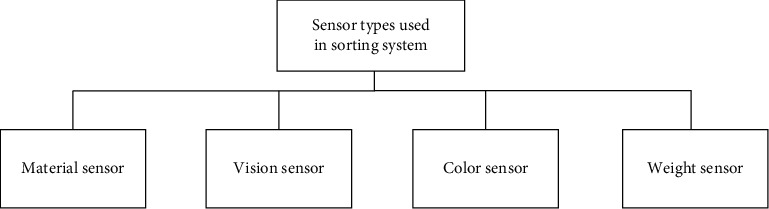
Sensor types that are used in sorting systems.

The sorting system is also used for sorting lima beans or sorting the connected characters of multiline license plates [15, 16]. The automated sorting system based on vision detection for fruit functions is based on the detection of the size and the shape of fruit in real-time [17, 18]. 3D vision sorting systems detect the object and pick it up using the robot arm, where the systems used two cameras to obtain 3D detection [19].  Figure 1 shows the types of sensors used in sorting systems in different applications.

Ilas et al. [20] explored different types of algorithms in order to find the possible trajectories of an autonomous robot moving through different types of obstacles. Based on the developed approach, the distance between the robot and the obstacles was determined by taking into account the width of the robot. It was found that the optimal trajectory can be chosen to plan the robot. The most significant advantages of the presented algorithms are the height accuracy and simplicity even when the resources for the calculations are at a medium level.

Benicky et al. [21] presented a developed technique based on the real-time aspect. The partial solution was found to detect fast movement using different algorithms such as corner detection and edge detection. The authors presented the way to classify the motion. Furthermore, the optimal and effective forms were found by applying the corner and edge detection methods that can be used in the applications of redundant data. They recommended to filter the redundant data (enter image) that will be used in the real-time analysis.

Alexander and Thathan [22] presented a developed control circuit that can be used for the solar-fed cascaded multilevel inverter for the 3-kW solar plant. The switches of the semiconductor of the multilevel inverter were reduced by decreasing the total harmonic distortion that leads to enhanced output power. Based on the developed system, the level of the output voltage was increased without using the output transformers of boost converters. MATLAB/Simulink was used to simulate the developed system under different working circumstances. The obtained results from Simulink agreed completely with the experimental results. It was also found that the presented inverter is more optimal for the grid-connected and standalone systems.

Table 1 shows the most available sensors and methods that are used for different types of sorting operations. Based on the available literature that used the camera with edge detection, Hough line transform was used to detect all line edges of the product and the cross-correlation method was used to scan all lines of template and product capturing image.

The main objective of this work is to develop a real-time sorting system for any specific product (complex shape) that can detect it from different products by using a camera and based on an image processing approach with minimum cost and time. The matching method is applied in this system at a speed that is calculated according to the characteristics of a camera such as the number of the frame. Increasing the characteristics of the selected camera will increase the system performance and accuracy. The developed system can match the target product with high accuracy and in different positions and orientations. The main contribution of this work was building a new sorting system with cost and high accuracy to sort any complex product/object with an acceptable time. Based on the available literature, it was found that most of the researchers used or developed complex and expensive sorting systems, which is considered an economic disadvantage from the view of the industrial sector.

### 1.1. Description and Working Principle of Sorting System

**Figure 2:**
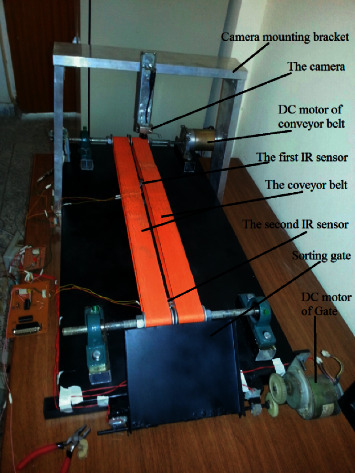
The main parts of the developed sorting system.

The developed sorting system consists of a conveyor belt that is connected to a DC motor, to handle the products to detect. Below this conveyor belt, two IR sensors were fixed, where the first sensor is to detect the arriving product under the camera to send the signal trigger to the camera. The camera is fixed above the conveyor belt to capture the images in real-time for the products. The second IR sensor is located before the sorting gate to detect the arrived target product. The gate is connected to the second DC motor to change the path of the target product when the target product arrives at the second IR sensor, then the sorting gate will be opened.  Figure 2 illustrates the details of the elements of the developed sorting system.

**Figure 3:**
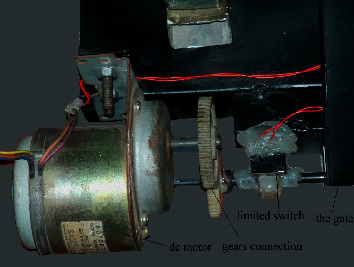
The sorting gate subsystem.

The microcontroller is the control unit in the system that connects with the program platform to transform input and output control signals. The optical encoder connects to the shift of the conveyor belt to calculate the speed of the belt which must be in the range of the frame of the camera. The sorting gate subsystem consists of a gate connected to a DC motor with a limited switch to open the gate at the suitable gap that is best to cross the desired product. Then, the gate closed after a few times need to cross the product as shown in Figure 3.

### 1.2. Theoretical Methodology of the Target Product Matching

The template image of the target product is prepared to match the color-capturing image for all products. The Canny edge detection method was applied to detect the desired line. The Canny strategy activity comprises six steps for the edge detection [23]. The gradient image *P*(*x*, *y*) is calculated from the partial derivatives in the *x-*direction and *y-*direction of the 2D image as

(1) $$\begin{array}{c} \nabla P\left(x,y\right)=\left[\frac{\partial P\left(x,y\right)}{\partial x},\frac{\partial P\left(x,y\right)}{\partial y}\right]. \end{array}$$

The measure pixel intensity rate at *x*-direction as

(2) $$\begin{array}{c} \frac{\partial P\left(x,y\right)}{\partial x}\approx \frac{P\left(x+0.5\Delta x,y\right)-P\left(x-0.5\Delta x,y\right)}{\Delta x}, \end{array}$$

and the measured pixel intensity rate at *y*-direction as

(3) $$\begin{array}{c} \frac{\partial P\left(x,y\right)}{\partial y}\approx \frac{P\left(x,y+0.5\Delta y\right)-P\left(x,y-0.5\Delta y\right)}{\Delta y}. \end{array}$$

The gradient magnitude can be found from

(4) $$\begin{array}{c} \left\|\nabla P\right\|=\sqrt{\frac{\partial P^{2}}{\partial x}+\frac{\partial P^{2}}{\partial y}}. \end{array}$$

Now, the Image Processed Hough Lines for Path Planning Application is applied to find the target line from capturing the image and detect its coordinate. The Hough transform obtained from the polar equation is

(5) $$\begin{array}{c} r=x\;\cos\;\theta +y\;\sin\;\theta , \end{array}$$

Here, *r* is the length of the line from the inception to the nearest point, and *θ* is the angle between the *x*-axis and the line associating the cause with that nearest point. It is subsequently conceivable to connect with each line of the picture, a couple (*r*, *θ*). The (*r*, *θ*) is the plane alluded as Hough space for the arrangement of straight lines in the two measurements. This portrayal causes the Hough to change reasonably exceptionally near the two-dimensional Radon change. The Hough change is numerically identical to the Radon change, yet the two changes have distinctive computational understandings customarily connected with them. Given a solitary point in the plane, at that point, the arrangement of every single consecutive line experiencing that point relates to a sinusoidal bend in the (*r*,*θ*) plane, which is novel to that point. A lot of at least two focuses that structure a straight line will create sinusoids which cross at the (*r*,*θ*) for that line. Hence, the issue of identifying collinear focuses can be changed over to the issue of finding simultaneous bends. The computational appeal of the Hough change emerges from subdividing the *r* parameter space into supposed accumulator cells, where [*r*_min_, *r*_max_] and [*θ*_min_, *θ*_max_] are the normal scopes of the parameter esteems. Normally, the most extreme scope of qualities is

(6) $$\begin{array}{c} -B\leq r\leq B\wedge -90\leq \theta \leq 90, \end{array}$$

where *B* is the most distant separation between inverse corners in the image. The cell at organizes (*i*, *j*) with aggregator esteem A (*i*, *j*) relates to the square connected with parameter space arranges (*r*_*i*_, *θ*_*j*_). At first, these cells are set to zero. At that point, for each nonfoundation point (*x*_*k*_, *y*_*k*_) in the picture plane (i.e., the *xy*-plane), it was assumed that *θ* is equivalent to every one of the permitted subdivision values on the *θ*-axis, where it explains the relating *p* that utilizing the condition *r* = *x*_*k*_ cos *θ+ y*_*k*_ sin *θ*.

The subsequent *R*-values are adjusted in the next step to the closest permitted cell esteem along the *r*-axis. The comparing accumulator cell is increased as well. In order to finish the process of this methodology, an estimation of *P* value in the cell was performed. An (*i, j*) implies that *P* focuses in the *xy*-plane that lies on hold *x* cos *θj* *+* *y* sin *θj* = *r*_*i*_. The quantity of subdivisions in the *rθ*-plane will be decided by the accuracy of the collinearity of these focuses. The aggregator cluster is alluded to the tool kit as the Hough transform matrix, or basically as the Hough transform.

An improved Hough transform (HT) technique is proposed to recognize line portions in images with entangled foundations. This work concentrates around distinguishing line sections of particular lengths, absolutely autonomous of earlier information on the first image [24]. The statistical method depends on the Hough transforms of line fragment location by taking about quantization error, picture clamor, pixel unsettling influence, and pinnacle spreading, likewise considering the decision of the facilitate starting point [25, 26]. Improvement of image processing by the changed Hough transformation applied for seam following the framework in gas metal arc (GMA) welding [27]. An improved Hough algorithm for line detection, which shares the comparative qualities of the modified Hough transform (MHT) and the Windowed random Hough transform (RHT) [28]. Hough transform (HT) has discovered monstrous viable applications in vision issues such as object detection, movement detection, biometric validation, medical imaging, remote information processing, and robot route [29]. Applying Hough transform formulation depends on the utilization of the inverse Radon operator, which decides the location and orientation of the lines in the image to the noise information image [30].

A matching technique consolidated Hough transform and Random sample consensus (RANSAC), which changes the issue of finding the right pixel-wavelength matching pairs into finding the privilege direct model [31]. A productive algorithm for line discovered from LRS information utilizing a novel jumping focuses singular value decomposition (SVD) and Hough transform-based transform, in which SVD is applied to irregular LRS focuses to accelerate the transform [32]. A modified amassing plan for the Hough transform makes it appropriate for computer frameworks with little, however, quick read-write memory [33]. Real-time lane detection for a driving system that uses edge detection and Hough transform has been created to help a driver in the path takeoff basic leadership, in order to decrease lost fixation and forestall a mishap while driving [34].

The cross-correlation method is applied to scan the pixel of the template and capture images to find the similarity between them. The cross-correlation is calculated as

(7) $$\begin{array}{c} \left(f.g\right)\left(\delta \right)=\int_{-\infty }^{\infty }\bar{f\left(t-\delta \right)}g\left(t\right)dt, \end{array}$$

where the $\bar{f\left(t\right)}$ is the complex conjugated for the *f (t)* that is represented the function of pixels.

### 1.3. Experiment of the Developed Sorting System

**Figure 4:**
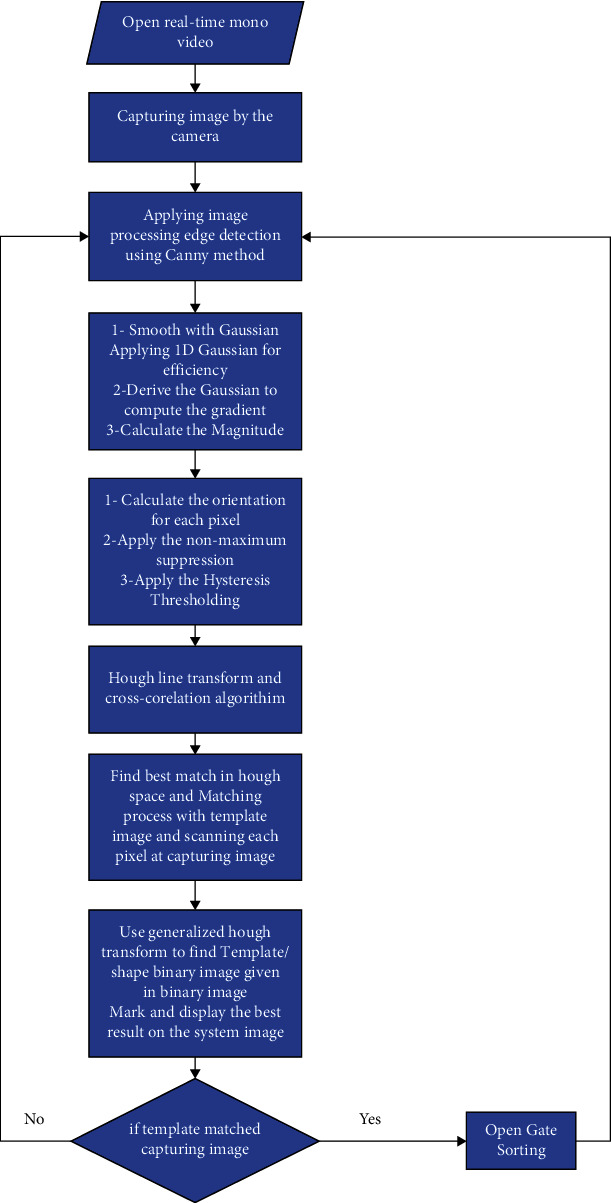
Flowchart showed the step of the system process.

**Figure 5:**
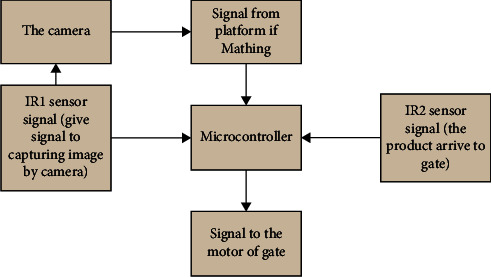
The main elements in the control unit of the sorting system.

The starting step of the sorting system is capturing pictures of the target product. Image processing Canny edge detection, Hough line transform, and cross-correlation are applied in the developed sorting system. The next step is the matching process between the image capturing and the templet of the selected product.  Figure 4 shows the flowchart of the main steps of the process achieved in the developed sorting system. The matching operation will be scanned pixels of templet and then compare them with pixels of capturing images. The comparison is at the edge of the product that detects in the template image with the edge that detects in the capturing image. If the matching is obtained, the gate will be opened to change the path of the selected product. The microcontroller is the center of the control unit of this system, where the signal that came from the IR1 sensor gives the signal to the microcontroller, and then it gives the signal to capture the new image. If matching happened, the platform gives the signal to the microcontroller and waits for the signal from the IR2 sensor to give the motor of the gate a new signal to open it.  Figure 5 demonstrates the block diagram of the control unit in the developed sorting system.

**Figure 6:**
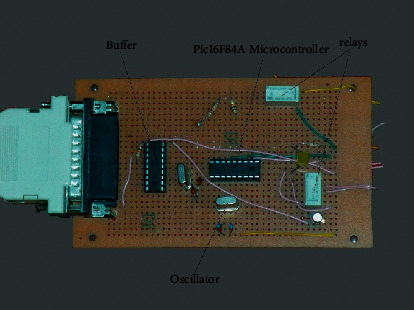
The microcontroller circuit.

The microcontroller circuit consisted of a (pic16f84a) microcontroller and two relays to control the sorting gate motor with a buffer that regulates the signal that comes from the software platform (Figure 6). The system was designed and implemented at a low-cost component with high sorting efficiency in real-time.

## 2. Results of the Sorting System and Discussions

**Figure 7:**
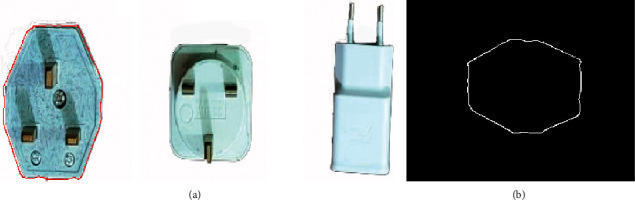
The matching of sample 1: (a) product image captured; (b) template image.

**Figure 8:**
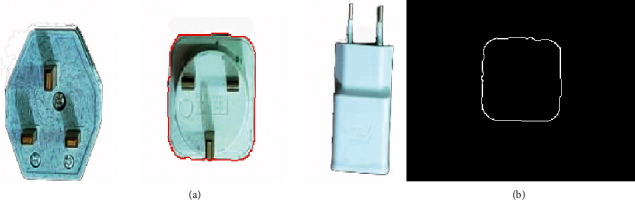
The matching of sample 2: (a) product image captured; (b) template image.

**Figure 9:**
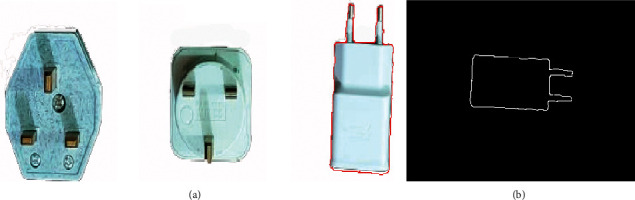
The matching of sample 3: (a) product image captured; (b) template image.

The automated industrial sorting system results are the decision to choose the target product from any product. The template image was obtained from edge detection for the target image of the product. The system scans all pixels capturing the image to detect the matching with the template image (Figures 7, 8 and 9) and shows the results of product detection.

The results show the template edge is converted to the red line and finds the edge of the product; then, the system tracks this product until arrived at the IR2 sensor. The template has a different orientation compared to the product, but the system has the ability to scan the pixel of the capturing image for the product to apply the matching process. The template is basically the image of the product that was taken in the early stage. In the next step, the image processing edge detection was applied with filtering to obtain this image edge templet. Any product that needs to sort it will call up the template and start the process of the system.

In the experimental work, three different samples of products were used to examine the accuracy of the sorting operation. The developed system can be sorted these three different shapes of product but with different time speeds of detection. The time of detection (matching image processing) is a function of the product shape, where the detection time increases if the geometry of the product has a complex shape and decreases if the geometry of the product has a simple shape. It was achieved 100 tests for each sample of selected products in order to explore the accuracy of sorting and the average time of detection for each product as shown in Table 2. It was found that the lowest time (6 s) needed for detection was for sample 2 and the highest one was for sample 3 (10 s). The reason behind such results is the shape of the sample which contains a number of lines and curvatures that affect significantly the sorting process.  Table 3 shows the accuracy of detection of the developed sorting system for the same samples. It was found that the highest accuracy (99%) was obtained for samples 1 and 2, and the lowest accuracy (98%) was obtained for sample 3.

The results of the developed sorting system are used to detect the target product by capturing an image at any position or orientation. One of the reasons to appear such kind of error is the lack of clarity for capturing the image that makes the edge of the product not distinguished. It can be considered the vibration of the camera or product is one of the main sources that reduces the stability of capturing the images used in the matching process.

**Figure 10:**
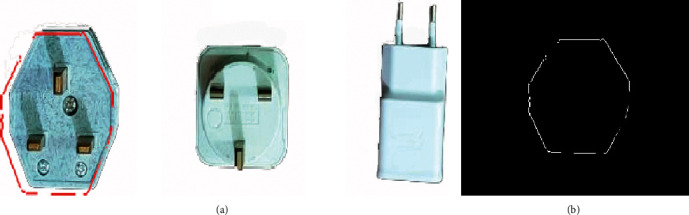
The matching of Prewitt edge method: (a) product image captured; (b) template image.

**Figure 11:**
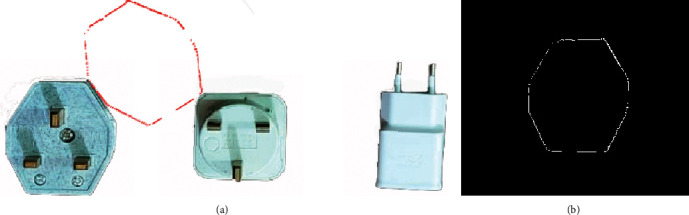
The matching of Roberts edge method: (a) product image captured; (b) template image.

**Figure 12:**
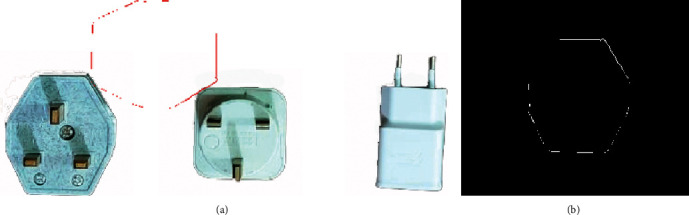
The matching of Sobel edge method: (a) product image captured; (b) template image.

In this research paper, other techniques of edge detection were explored in order to verify the selected technique and show the advantages of the selected approach compared with other available approaches. Figures 10–12 show the results of sorting based on other edge techniques, where Prewitt, Roberts, and Sobel techniques were selected. Based on the obtained results, existing errors can be noticed to detect the desired product. The Prewitt method finds the product but not in the correct position, and such a case will lead to an inaccurate sorting process and affect the overall performance of the system as shown in Figure 10.

On the other hand, Roberts and Sobel's methods cannot find the desired product and the position was not correct, and then the sorting gate was opened at the wrong time as shown in Figures 11 and 12. The reason for incorrect detection of the products is the detecting process does not cover the entire template of the outer edge for the desired product. The results of the image processing program of the system will give the wrong boundary of pixels in the template and the product image captured. Based on the all obtained results, it was proved that the optimal method is Canny which is used in this system that has the ability to find the optimal outer edge of the product and obtain high-performance sorting.

## 3. Conclusions and Remarks

In this paper, a new automated sorting system was designed and implemented that has the ability to sort any product in real-time. The system can detect the product at any position or orientation and determine the edge of the target product. The template image was prepared out of the processing of the system and changes with changing the type of product. It was found that the time of processing is different, and it is a function of the size of the capturing image and the volume of the detecting product. One of the disadvantages that lead to an increase in the time and decrease in the accuracy of the detections is the low resolution of the used camera. The captured image at a high-speed conveyer belt increased the error in the sorting system because of distorting the image, where the matching process needs sufficient time for detection. The main advantages of the new sorting system are the ability to sort any product with different shapes, even if it is complex in shape. The complex shape products have sharp edges and a lot of curves, for example, sample no. 3 and gears. The limitation of this system is the similar out edge of products like they are all circular or square, etc. Also, there is another important point that should be mentioned which is the suitability of the illumination, where it should be with suitable intensity and distance from the conveyor belt to capture the image clearly.

One of the most important points that will be discussed in detail in the subsequent research is how to sort products that are completely identical in their external borders but contain holes and cavities in different locations and sizes. Such kind of sorting needs to study the internal details of the object/product, such as the locations and sizes of the holes, and how to sort it in any orientation.

## Figures and Tables

**Table 1:** The sensors and methods that are used in sorting operations.

No.	Classifications of sensors and methods	Type of sorting
1	Camera sensor [2, 3]	Color, size, shape
2	LIDAR [1]	Size
3	Heuristic method [1]	Shape
4	Oscillator frequency [4]	Material types
5	Histogram + SVM [6]	Object properties
6	Deep learning + CCD sensor [8]	Object properties
7	Municipal solid waste + SVM [10]	Recyclable materials
8	Camera + weight sensor + silhouette algorithm [11]	Defect detection and remanufacturing
9	Camera + fuzzy logic algorithm [12]	Defect detection and classification
10	HMI (human-machine interface) [14]	Color box

**Table 2:** The average time needed for the detection of the samples.

Product sample no.	No. of experiment	Rate of time detection (s)
Sample no. 1	100	8
Sample no. 2	100	6
Sample no. 3	100	10

**Table 3:** The accuracy of the detection of the samples.

No. Of product sample	No. of experiment	The number of successful detection
Sample no. 1	100	99
Sample no. 2	100	99
Sample no. 3	100	98

## Data Availability

No data were used to support the study.
